# Cross-cultural adaptation of the Beliefs about Medicines Questionnaire into Portuguese

**DOI:** 10.1590/S1516-31802013000100018

**Published:** 2013-04-01

**Authors:** Teresa Salgado, Alexandra Marques, Leonor Geraldes, Shalom Benrimoj, Robert Horne, Fernando Fernandez-Llimos

**Affiliations:** I MSc. Pharmacist, Research Institute for Medicines and Pharmaceutical Sciences (iMed. UL), Faculty of Pharmacy, Universidade de Lisboa, Lisbon, Portugal.; II MSc. Pharmacist, Faculty of Pharmacy, Universidade de Lisboa, Lisbon, Portugal.; III PhD. Head of School of Pharmacy Graduate School of Health, University of Technology Sydney, Sydney, Australia.; IV PhD. Professor and Director of the Centre for Behavioural Medicine, UCL School of Pharmacy, University College London, London, United Kingdom.; V PhD. Assistant Professor, Faculty of Pharmacy, Universidade de Lisboa, Lisbon, Portugal.

**Keywords:** Psychometrics, Questionnaires, Medication adherence, Reproducibility of results, Portugal, Psicometria, Questionários, Adesão à medicação, Reprodutibilidade dos testes, Portugal

## Abstract

**CONTEXT AND OBJECTIVES:**

The Beliefs about Medicines Questionnaire (BMQ-Specific) has proven useful for measuring patients’ beliefs and associating them with non-adherence to treatment in several illness groups. The aim was to cross-culturally adapt the BMQ-Specific into Portuguese for the general population of medicine users.

**DESIGN AND SETTING:**

Cross-sectional study conducted among users of public hospitals and outpatient clinics in Guarda and Covilhã, Portugal.

**METHODS:**

The BMQ-Specific was translated using international recommendations for performing cross-cultural adaptation and was administered to 300 patients. An initial principal component analysis (PCA) was conducted with the extraction criterion of eigenvalue > 1.0, followed by a second PCA with restriction to two components. Reliability was assessed by calculating Cronbach’s alpha coefficient.

**RESULTS:**

The mean scores obtained for the Necessity and Concerns subscales of the Portuguese BMQ-Specific were 19.9 (standard deviation, SD = 2.8) (range 10 to 25) and 17.7 (SD = 3.9) (range 6 to 30), respectively. The first PCA produced an unstable three-component structure for the Portuguese BMQ-Specific. The final PCA solution yielded a two-component structure identical to the original English version (a five-item Necessity and a six-item Concerns subscale), and explained 44% of the variance. Cronbach’s alpha for the complete Portuguese BMQ-Specific was 0.70, and 0.76 and 0.67 for the Necessity and Concerns subscales, respectively.

**CONCLUSION:**

A cross-culturally adapted Portuguese version of the BMQ-Specific questionnaire for use among the general population of medicine users was obtained, presenting good internal consistency and component structure identical to the original English version.

## RESUMO

**CONTEXTO E OBJETIVO:**

O Beliefs about Medicines Questionnaire (BMQ-Específico) tem se mostrado útil na mensuração sobre as crenças dos pacientes e associando-os à não adesão ao tratamento em vários grupos de doença. O objetivo foi realizar a adaptação transcultural do BMQ-Específico em Português para a população geral de usuários de medicamentos.

**TIPO DE ESTUDO E LOCAL:**

Estudo transversal conduzido com usuários de hospitais públicos e clínicas ambulatoriais em Guarda e Covilhã, Portugal.

**MÉTODOS:**

O BMQ-Específico foi traduzido utilizando-se recomendações internacionais para realizar a adaptação transcultural e foi administrado a 300 pacientes. Uma primeira análise de componentes principais (ACP) com critério de extração *eigenvalue* > 1.0 foi realizada, seguida de uma segunda ACP com restrição para dois componentes. A confiabilidade foi avaliada calculando-se o coeficiente alfa de Cronbach.

**RESULTADOS:**

Os escores médios obtidos para as subescalas de Necessidade e Preocupação do BMQ-específico em Português foram de 19,9 (desvio padrão, DP = 2,8; intervalo 10 a 25) e 17,7 (DP = 3,9; intervalo 6 a 30), respectivamente. A primeira ACP produziu estrutura instável de três componentes para o BMQ-específico em português. A solução final de ACP produziu estrutura de dois componentes idênticos à versão original em inglês (subescala de Necessidade de 5 itens e subescala de Preocupação de 6 itens), explicando 44% da variância. Alfa de Cronbach para o BMQ-Específico em Português completo foi de 0,70, sendo 0,76 e 0,67 para as subescalas de Necessidade e Preocupação, respectivamente.

**CONCLUSÃO:**

A versão adaptada do questionário BMQ-Específico em português para ser utilizada na população em geral dos usuários de medicamentos foi obtida, apresentando boa consistência interna e estrutura do componente idêntica à versão original em inglês.

## INTRODUCTION

The World Health Organization has recognized non-adherence to long-term therapies as a “worldwide problem of striking magnitude” averaging 50% in developed countries.^1^ Evidence from several studies has shown the impact of non-adherence in terms of poor health outcomes and higher healthcare costs relating to conditions such as diabetes,^2^^,^^3^^,^^4^ hypertension^5^^,^^6^ or asthma.^7^

Adherence is determined by the interplay of a multitude of factors, of which some are patient-related and include patients’ resources, knowledge, attitudes, beliefs, perceptions and expectations towards medication.^1^ Treatment adherence appears to depend primarily on patients’ beliefs about treatment benefits and, to a lesser extent, on sociodemographic and clinical factors.^8^^,^^9^^,^^10^ Intentional non-adherence appears to be related to patients’ beliefs and their motivation to take the prescribed medication, whereas unintentional non-adherence has to do with patients’ skills or ability to take that medication (e.g. forgetfulness or manual dexterity).^11^ Intentional non-adherers hold beliefs significantly different from those of adherers and unintentional non-adherers.^12^

The Beliefs about Medicines Questionnaire (BMQ) was created to respond to a need for practical measurements on commonly held beliefs about medication. In addition, it aimed to assess the nature of beliefs about medications, the distribution of these beliefs among different populations and the relationships between beliefs about medicines, beliefs about illnesses and adherence behavior. The questionnaire was developed based on beliefs identified in the literature that appeared to be common to patients with a range of chronic illnesses, and based on interviews conducted with patients receiving regular medication for chronic illnesses. The questionnaire was validated among a sample of asthmatic, diabetic, renal, cardiac, psychiatric and general medical patients. The final version of the BMQ is composed of two sections: the General section (BMQ-General), which assesses more general beliefs about medicines and includes the General-Harm and the General-Overuse subscales; and the Specific section (BMQ-Specific), which explores beliefs about particular medication and comprises the Specific-Necessity and Specific-Concerns subscales.^13^

The BMQ-Specific section was designed to put into practice the Necessity-Concerns framework in an attempt to predict patients’ adherence, based on their beliefs about their personal need for the treatment versus their concerns regarding potential adverse effects. Several studies have shown the usefulness of the Necessity-Concerns framework for correlating patients’ beliefs and treatment non-adherence across a range of chronic illness groups: diabetes,^14^ hypertension,^15^ asthma,^16^ cystic fibrosis,^17^ depression,^18^ mental illness,^19^ bipolar disorder,^20^ inflammatory bowel disease,^21^ HIV/AIDS,^22^ rheumatoid arthritis,^23^ renal disease, cardiac disease and cancer.^10^ Not only has the BMQ-Specific been useful in providing insight on patients’ intentions to take medication, but also it has been informative regarding patients’ actual medication-taking behavior.^16^ Additionally, the short administration time makes the BMQ-Specific a potentially applicable instrument in clinical practice.

The BMQ has been adapted and used in several countries,^16^^,^^24^^,^^25^^,^^26^^,^^27^ but no cross-cultural adaptation into Portuguese has been performed to date. Therefore, the aim of this study was to cross-culturally adapt the Specific section of the Beliefs about Medicines Questionnaire (BMQ-Specific) into Portuguese for the general population of medicine users.

## METHODS

The BMQ-Specific is an eleven-item questionnaire that comprises two subscales: a five-item Necessity scale, to assess beliefs about the necessity for prescribed medication (Specific-Necessity), and a six-item Concerns scale, to assess beliefs about the danger of dependence and long-term toxicity and the disruptive effects of medication (Specific-Concerns).^13^ Each item is scored on a five-point Likert scale (1 = strongly disagree, 2 = disagree, 3 = uncertain, 4 = agree and 5 = strongly agree), and the total scores for the Necessity and Concerns subscales range from 5 to 25 and from 6 to 30, respectively. The higher the score is, the greater the patient’s belief in the concept represented by the scale is. A necessity-concerns differential can also be calculated by subtracting the Concerns subscale scores from the Necessity subscale scores, such that higher differential scores indicate higher perceived necessity and/or lower concerns, thereby representing lower likelihood of intentional non-adherence.^12^

### Cultural adaptation

Written authorization to translate the original English version of the BMQ-Specific into Portuguese was obtained from Prof. Robert Horne at the School of Pharmacy, University of London. Prof. Horne requested that the adaptation should be done on the modified version of the BMQ-Specific, given that it has been subjected to refinements since its original publication in 1999 to better respond to problems resulting from its administration.

The translation process was conducted based on the Principles of Good Practice for Translation and Cultural Adaptation.^28^ In the first translation step, an independent hospital pharmacist who was a native Portuguese speaker and fluent in English, and who was aware of the objectives of the study, translated the original version of the questionnaire into Portuguese. The translated version was then discussed with another member of the Portuguese branch of the research team and discrepancies were reconciled. The translated version was then back-translated into English by a native English speaker who was fluent in Portuguese (an English-language professor), from whom both the objectives of the study and the original version of the questionnaire were concealed. The back-translated and original versions of the BMQ-Specific were compared by Prof. Robert Horne and his team, and subsequent discrepancies were resolved via e-mail correspondence between the Portuguese and English research teams. The forward translation was then finalized based on the preceding discussion and a conceptual, semantic and operational version^29^ of the Portuguese BMQ-Specific was thus obtained.

### Participants and recruitment

The study was conducted in two different cities in Portugal (Guarda and Covilhã) between March and June 2010. Ethical approval was granted by two ethics boards: the Ethics Committee of the General Hospital of Cova da Beira, and the Ethics Committee of the Local Health Unit of Guarda. The authorization included the above mentioned sites, as well as subsidiary outpatient clinics. Patients in an external consultation waiting room were approached by a member of the research team and were invited to participate. Informed written or verbal consent in accordance with the requirements of each committee was obtained from individuals who agreed to participate in the study.

The sample size was calculated based on a minimum subject-to-item ratio of 20:1 (20 x 11 = 220), since it had previously been shown that larger samples tend to produce more accurate solutions.^30^ However, the Portuguese version of the BMQ-Specific was ultimately administered to a sample of 300 participants (150 in Guarda and 150 in Covilhã), which is the minimum required for good adequacy of sample size according to some authors.^31^

Participants over 18 years of age who answered affirmatively to the question “Do you frequently take medicines?” were included in the study. To reduce the risk of bias, participants were informed that the questionnaires would be kept confidential and anonymous, and that the results would not be seen by healthcare providers, thus not affecting the quality of care received. Sociodemographic data such as age, gender, level of education and origin (rural/urban) were also collected. The questionnaire was administered by a member of the research team in the form of a structured interview.

### Data analysis

Descriptive statistical analyses were used on the sociodemographic data. An exploratory principal component analysis (PCA) with non-orthogonal rotation (oblimin with Kaiser normalization) was performed to examine the number of components in the Portuguese version of the BMQ-Specific.^32^ The criterion for component extraction was an eigenvalue > 1.0 (Kaiser’s criterion). Since the original English version presents a two-component structure, a subsequent PCA was conducted with restriction to two components. Cronbach’s alpha coefficient was calculated to assess the internal consistency of the Portuguese version of the BMQ-Specific. A Cronbach’s alpha value over 0.70 indicated acceptable internal consistency.^33^ The statistical analyses were performed using the Statistical Package for the Social Sciences (SPSS), version 16.

## RESULTS

### Sample characteristics

Among the 300 participants enrolled in the study, 69.7% were female and 52.3% originated from an urban area. The mean age of the respondents was 62.0 years (standard deviation, SD = 14.4) (range 18 to 94) and their level of education was as follows: 9.7% were unable to read or write, 8.0% were able to read and/or write but had not had any formal education, 51.3% had completed elementary school, 20.0% had completed high school and 11.0% had had higher education.

The mean scores obtained for the Necessity and Concerns subscales of the Portuguese BMQ-Specific were 19.9 (SD = 2.8) (range 10 to 25) and 17.7 (SD = 3.9) (range 6 to 30), respectively. The mean total score for the BMQ-Specific was therefore 37.6 (SD = 5.2); the minimum score obtained was 22 and the maximum was 54, out of the possible total of 55.

### Principal component analysis

The Kaiser-Meyer-Olkin value was calculated as 0.75, which indicated that PCA on this dataset was feasible. The chi-square obtained for Bartlett’s test of sphericity was 617.8 (significance level < 0.001), which meant that the correlation matrix was not an identity matrix and appeared to be factorable.^32^ The correlation matrix obtained showed that only the items N2 and N3 presented correlations over 0.500 (Pearson correlation coefficient = 0.603).

**Figure 1.**
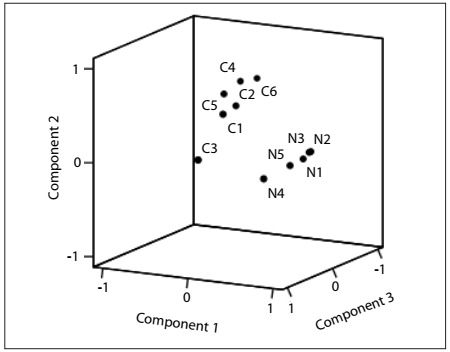
Component plot for first principal component analysis (criterion for component extraction: eigenvalue > 1.000).

The exploratory PCA, which was conducted using the non-orthogonal (oblimin with Kaiser normalization) method of rotation, revealed a preliminary three-component structure explaining 53.5% of the variance. As depicted in Table 1, the first component included items N1 to N5, the second component comprised items C1, C2, C4, C5 and C6, and item C3 appeared isolated in a third component. Nevertheless, this third component presented an eigenvalue = 1.007, i.e. close to the predefined extraction criterion (eigenvalue > 1.000). The items included in the first component presented high component loadings, ranging from 0.577 to 0.784, and the same was found for the items in the second component, which had component loadings ranging from 0.566 to 0.722. The isolated item C3 presented lower loadings for the two previous components, respectively 0.086 and 0.202, but a very high loading in the third component (0.804). The component plot in rotated space (Figure 1) highlighted the first component marked by high loadings for the Necessity items, the second component marked by high loadings for the Concerns items and the third component comprising item C3, which appeared to be not very distant from the items in the second component (Concerns).

**Table 1.** Exploratory principal component analysis with non-orthogonal (oblimin with Kaiser normalization) rotation

Items	Components		
	1	2	3
N2 - A minha vida seria impossível sem estes medicamentos *My life would be impossible without these medicines*	0.784	0.143	0.048
N3 - Sem estes medicamentos, eu estaria muito doente *Without these medicines I would be very ill*	0.784	0.142	0.020
N1 - Atualmente, a minha saúde depende destes medicamentos *My health, at present, depends on these medicines*	0.734	0.077	0.069
N5 - Estes medicamentos protegem-me de ficar pior *These medicines protect me from becoming worse*	0.671	0.041	0.191
N4 - A minha saúde no futuro dependerá destes medicamentos *My health in the future will depend on these medicines*	0.577	-0.012	0.483
C6 - Estes medicamentos dão-me desagradáveis efeitos secundários *These medicines give me unpleasant side effects*	0.131	0.722	-0.118
C4 - Estes medicamentos perturbam a minha vida *These medicines disrupt my life*	0.038	0.721	0.014
C5 - Às vezes, preocupo-me em ficar demasiado dependente destes medicamentos *I sometimes worry about becoming too dependent on these medicines*	0.016	0.669	0.255
C2 - Às vezes, preocupo-me com os efeitos a longo prazo destes medicamentos *I sometimes worry about long-term effects of these medicines*	0.164	0.586	0.289
C1 - Preocupa-me ter de tomar estes medicamentos *Having to take these medicines worries me*	0.154	0.566	0.491
C3 - Estes medicamentos são um mistério para mim *These medicines are a mystery to me*	0.086	0.202	0.804

Criterion for component extraction: eigenvalue > 1.0.

**Figure 2.**
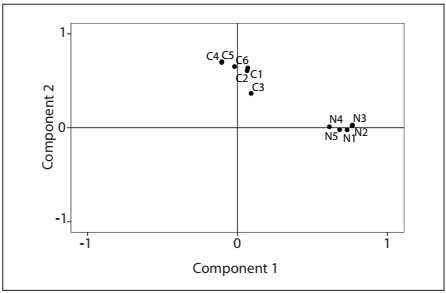
Component plot for second principal component analysis (criterion for component extraction: eigenvalue > 1.007).

A second PCA with restriction to two components was then conducted to respect the original structure of the BMQ-Specific. The criterion for component extraction in the second analysis was an eigenvalue > 1.007. The total variance explained in the two-component solution was 44.4%. In this new structure, the previously isolated C3 item was now included in the second component, thus making the Portuguese BMQ-Specific structurally identical to the original questionnaire (Table 2). The loadings of items included in the first component ranged from 0.615 to 0.772, whereas the loadings of items in the second component ranged from 0.383 to 0.683. The component plot in rotated space (Figure 2) showed a robust final solution of two components, in which Necessity items had high loadings in the first component while Concerns items had high loadings in the second component. Item C3 was clearly shown to be part of the Concerns component, and appeared to be sufficiently distant from the Necessity component.

**Table 2.** Principal component analysis with restriction to two components

Items	Components	
	1	2
N2 - A minha vida seria impossível sem estes medicamentos *My life would be impossible without these medicines*	0.772	0.173
N3 - Sem estes medicamentos, eu estaria muito doente *Without these medicines I would be very ill*	0.769	0.165
N1 - Atualmente, a minha saúde depende destes medicamentos *My health, at present, depends on these medicines*	0.726	0.115
N5 - Estes medicamentos protegem-me de ficar pior *These medicines protect me from becoming worse*	0.677	0.108
N4 - A minha saúde no futuro dependerá destes medicamentos *My health in the future will depend on these medicines*	0.615	0.126
C5 - Às vezes, preocupo-me em ficar demasiado dependente destes medicamentos *I sometimes worry about becoming too dependent on these medicines*	0.028	0.683
C4 - Estes medicamentos perturbam a minha vida *These medicines disrupt my life*	0.024	0.674
C1 - Preocupa-me ter de tomar estes medicamentos *Having to take these medicines worries me*	0.188	0.649
C6 - Estes medicamentos dão-me desagradáveis efeitos secundários *These medicines give me unpleasant side effects*	0.103	0.647
C2 - Às vezes, preocupo-me com os efeitos a longo prazo destes medicamentos *I sometimes worry about long-term effects of these medicines*	0.178	0.619
C3 - Estes medicamentos são um mistério para mim *These medicines are a mystery to me*	0.160	0.383

### Reliability

Using Cronbach’s alpha coefficient, the internal consistency of the BMQ-Specific was 0.700 overall, while Cronbach’s alphas for the Necessity and Concerns subscales were 0.757 and 0.665, respectively. If item C3 was deleted, Cronbach’s alpha for the Concerns subscale would increase to 0.678, but the complete BMQ-Specific alpha would decrease to 0.698. Deletion of one of the items N2 or N3 would result in reduction of the BMQ-Specific alpha from 0.700 to 0.671 or 0.672, respectively.

## DISCUSSION

To the best of our knowledge, this is the first study describing cross-cultural adaptation of the Portuguese version of the BMQ-Specific, a “flexible instrument which can be adapted to assess beliefs about all medicines for a particular condition or for individual components of the regimen”.^13^

The high response rate to the questionnaire can be explained by our use of an interviewer-administered procedure. In a previous study in which the option of self-administration or interviewer-administration was available, only 10% of the participants chose to complete the BMQ independently.^24^ This may be even more relevant in a population in which 9.7% were illiterate and 59.3% did not begin high school. This level of education, along with the average age of the participants, is a common characteristic among people who regularly use medicines in Portugal.^34^

The mean scores for the Necessity and Concerns subscales of the Portuguese version of the BMQ-Specific (19.9 and 17.7, respectively) were comparable to what has been obtained using the original English version, in which the mean scores for the Necessity and Concerns subscales have ranged from 17.72 to 21.26 and from 12.91 to 15.75, respectively.^13^ The results reported from the German version were mean scores of 22.27 and 13.55 for the Necessity and Concerns subscales, respectively.^25^ The Portuguese results were similar to the English and German findings, although with a slightly lower Necessity-Concerns differential.

The initial solution produced by the exploratory PCA was discarded because it left item C3 (“My medicines are a mystery to me”) isolated from the other two groups of items. Components with fewer than three items are known to be weak and unstable.^30^ In the final PCA solution, item C3 became part of the second component (Concerns), even though it presented a low component loading (0.383). Issues with this item had previously been raised during the development of the original instrument, in which item C3 had a loading of 0.39 on the Concerns subscale during the confirmatory factor analysis, and an even higher loading on the Harm subscale (0.55) of the General section of the BMQ.^13^ This suggests that inclusion of item C3 in the questionnaire might not be appropriate. Indeed, the word “mystery” posed a challenge to the translation process into Portuguese, and this has also been reported for other languages like those of Scandinavia.^35^

The two-component final solution of the Portuguese version of the BMQ-Specific explained 44.4% of the total variance, which was slightly lower than the 51% variance reported in the original English version.^13^ This solution revealed a robust structure with clearly defined components, as can be seen in the component plot in rotated space (Figure 2).

The results provide good support for confirming the reliability of the Portuguese version of the BMQ-Specific. The Cronbach’s alphas for the Necessity and Concerns subscales (0.757 and 0.665, respectively) were in agreement with the values reported for the original English version, in which Cronbach’s alpha ranged from 0.55 to 0.86 and from 0.63 to 0.80 for the Necessity and Concerns subscales, respectively,^13^ as well as with the results obtained for the Italian version (0.78 for the Necessity and 0.72 for the Concerns subscales).^24^ However, all these values were lower than the Cronbach’s alphas attained for the German version (0.83 for both subscales).^25^

During the present analysis, two potential modifications to the BMQ-Specific could have been considered. Deleting item C3 would result in an increase in Cronbach’s alpha for the Concerns subscale from 0.665 to 0.678, but Cronbach’s alpha for the complete BMQ-Specific would decrease from 0.700 to 0.698. Deleting one of the items N2 or N3, which presented moderate correlations, would result in a reduction in Cronbach’s alpha for the complete BMQ-Specific from 0.700 to 0.671 or 0.672, respectively. Therefore, the slight gains obtained from any of these potential modifications would not justify the loss of the structural identicalness between the Portuguese and English versions of the questionnaire.

Our study presents some limitations. Since we used an interviewer-administered questionnaire, the cross-culturally adapted version should only be used this way. On the other hand, this is probably the best option for Portuguese medicine users. Although in-depth analysis was conducted on the construct validity of the original version of the BMQ, further analysis should be undertaken to determine whether the Portuguese version presents a correlation with medication adherence. To accomplish this goal, a reliable and valid instrument to evaluate adherence that has previously been adapted into Portuguese should be selected. With regard to future research, further work is warranted in order to investigate the relationship between beliefs and adherence among Portuguese-speaking patients who take medicines regularly, as well as to test the instrument on different populations and in different settings.

## CONCLUSION

A cross-culturally adapted Portuguese version of the BMQ-Specific questionnaire for use among the general population of medicine users was obtained. This version presented good internal consistency and component structure identical to the original English version.
